# Vaccination in immunocompetent and immunosuppressed youth with special health care needs during healthcare transition

**DOI:** 10.1016/j.clinsp.2026.101032

**Published:** 2026-07-08

**Authors:** Flavia C. Yarshell, Clara R. Doria, Ana Paula S. Ferrer, Artur F. Delgado, Claudia B. Fonseca, Darusa C. Souza, Pedro Henrique P. Sampaio, Maria Fernanda B. Pereira, Nadia E. Aikawa, Clovis A. Silva

**Affiliations:** a Instituto da Criança e do Adolescente, Hospital das Clínicas da Faculdade de Medicina da Universidade de São Paulo (HCFMUSP), São Paulo, SP, Brazil; b Rheumatology Division, Hospital das Clínicas da Faculdade de Medicina da Universidade de São Paulo (HCFMUSP), São Paulo, SP, Brazil

Vaccination represents one of the most effective, accessible, and cost-efficient strategies to reduce morbimortality associated with viral and bacterial infections in adolescents with chronic illnesses.^1^ particularly in Youth with Special Health Care Needs (YSHCN).^2^ The National Survey of Children’s Health (NSCH) recognizes YSHCN using five-item criteria. These criteria categorize teens with chronic conditions lasting12-months or longer who need increased medical, therapeutic, or educational services, use prescription drugs, and experience functional and developmental limitations.^2^

Of note, both inactivated and live attenuated vaccines are incorporated into routine childhood immunization programs worldwide for these conditions.^1^ In some chronic diseases, this care becomes even more critical, given the immunological vulnerability imposed by the disease itself or by the pharmacological agents used in its management. In this context, maintaining an up-to-date immunization schedule is widely recognized as a marker of quality of care for this population. Nevertheless, the vaccination in teens with chronic conditions, particularly YSHCN, remains a subject of ongoing discussion, particularly regarding the disease and vaccine safety, and humoral and cellular immunogenicity of certain vaccines in the setting of distinct disease manifestations and their corresponding treatments.^1^^,^^3^

Additionally, the rise of disability-adjusted life-years observed in recent decades across a broad spectrum of conditions has increased the demand for transition of these patients from pediatric to adult care.^4^ Healthcare Transition (HCT) is broadly defined as the planned movement of teens with chronic conditions from a pediatric to an adult model of health care. Evidence-based frameworks defined core principles to guide this transition process, grounded in effective communication with teens, promoting autonomy and self-management, and recognition of vulnerabilities of each patient.^5^

In this context, immunization practices represent a particularly relevant marker of care quality and clinical challenge during this transitional period. Nevertheless, vaccination outcomes among teens with chronic conditions, particularly YSHCN, undergoing HCT remains poorly characterized in literature.

To address this gap, a comprehensive search of the PubMed/MEDLINE database was conducted for articles published up to March2026. The search strategy was structured in three thematic blocks combined with the Boolean operator and: a)A healthcare transition block, anchored on the MeSH term “Transition to Adult Care” and complemented by free-text terms such as “healthcare transition”, “transitional care”, and “transfer to adult care”; b)A disease block, covering17clinical categories including specific medical specialties and their84chronic conditions among adolescents; and c)A vaccination block, encompassing terms related to vaccine coverage, uptake, hesitancy, and refusal. Each disease category was searched independently, and duplicated results were excluded. Moreover, these MeSH terms were applied to YSHCN and various chronic conditions during Healthcare Transition (HCT).

The search retrieved9articles, and only6 of them specifically evaluated vaccination during healthcare transition from pediatric to adult clinics. Among these6manuscripts, only Croucher et al., 2013.^6^ provided a cohort study of vaccine coverage among52perinatally infected with HIV patients attending a dedicated transition to adult care clinic, of whom34started hepatitis-B immunization, with23/34(67%) fully vaccinated, and 18/34(53%) vaccinated within6-months during transfer process. Benchimol et al., 2022.^7^ and Alwassief et al., 2025.^8^ mentioned the importance to assess vaccination status during the transition process, without specific analysis of coverage data. Silva et al., 2015.^9^ Brandt et al., 2022.^10^ and Gerber et al., 2024.^11^ studied different points of management chronic diseases, discussing the need of vaccination and transitional care in patients with childhood-onset systemic lupus erythematosus, cancer survivors and chronic kidney conditions, without examining their intersection. In addition, to the best of our knowledge, no studies have specifically investigated the role of vaccination support during the healthcare transition process for YSHCN with chronic conditions during HCT.

To guide clinical practice and research on this subject, familiarity with vaccination guidelines issued by national health authorities and disease control agencies is essential for immunocompetent and immunosuppressed YSHCN with chronic conditions during HCT. Given the complexity of immunization schedules in this population, often requiring multiple vaccines across different timepoints, an age-structured approach may be particularly beneficial, especially when aligned with the specific phase of HCT faced by the patient and the broader factors inherent to this process, such as readiness assessment and transition planning.

A significant framework to support this process is the six core elements of HCT personalized to different clinical practice settings and organized around three age-defined phases: 12to14years (early preparation), 14to18years (transition planning and readiness assessment), and 18to26years (transfer and integration to adult care). The main objective of this process is to promote self-management and autonomy, supporting young people to assume primary responsibility for their own health care decisions.^5^ Although vaccination should be addressed longitudinally across all HCT phases, the transition planning and readiness assessment phase (14‒18years) may represent a critical opportunity for comprehensive immunization review and catch-up strategies before transfer to adult care.

When guiding vaccination during HCT, the patient’s immune status deserves particular attention and must be carefully assessed, as indications differ substantially between immunocompetent and immunosuppressed individuals, especially regarding live-attenuated vaccines, which are strictly contraindicated in severe immunosuppression, as well as the optimal timing of non-live vaccines to improve immune responses. Remarkably, immunosuppression may be a characteristic of the underlying condition or a transient consequence of treatment, meaning that vaccination eligibility may change over time and should be reassessed at each clinical appointment. In addition, a comprehensive evaluation of immunization status should include verification of vaccine series indicated for earlier age groups that may remain incomplete. A catch-up opportunity that the HCT encounter is particularly well suited to address.

Table 1 includes recommended vaccines for immunocompetent and immunosuppressed YSHCN with chronic conditions according to the age groups during HCT.^12^, ^13^, ^14^, ^15^, ^16^, ^17^ This framework is recommended to support clinicians involved in HCT process, particularly for the vulnerable population of immunosuppressed YSHCN with chronic conditions. Finally, it is hoped that this framework may help as a practical resource for daily clinical practice and orientation for further studies investigating vaccine immunogenicity and safety, coverage, and adherence during HCT.

**Table 1:** Recommended vaccines for immunocompetent and immunosuppressed youth with special health care needs (YSHCN) and with chronic conditions according to the age groups during healthcare transition.^12^, ^13^, ^14^, ^15^, ^16^, ^17^   .

	Early preparation (12–14 years)	Transition planning (14‒18 years)	Transfer to adult care (18‒26 years)
**Immunocompetent (IC)**	Annual inactivated or live-attenuated influenza	Annual inactivated or live-attenuated influenza	Annual inactivated or live-attenuated influenza
	COVID-19 (shared clinical decision-making)[13]	COVID-19 (shared clinical decision-making)[13]	COVID-19 (shared clinical decision-making)[13]
	dTPa booster	MenACWY (booster)	Mpox^b^
	MenACWY		
**All IC age groups**	Verify and complete if incomplete series: 10/13/15/20- PCV, 23-PPSV, hepatitis A, hepatitis B (consider anti-HBs serology), MMR, varicella, yellow fever, dengue^a^, IPV, Hib, dTPa/dTP, MenC/ACWY, MenB, 9vHPV or 4vHPV. In pregnant adolescent: RSV, dTPA booster, inactivated influenza, COVID-19.		
**Immunosuppressed (IS)**^c^	Annual inactivated influenza	Annual inactivated influenza	Annual inactivated influenza
	COVID-19 (shared clinical decision-making)[13]	COVID-19 (shared clinical decision-making)[13]	COVID-19 (shared clinical decision-making)[13
	dTPa booster	MenACWY (booster)	
	MenACWY		
**All IS age groups**	Verify and complete if series incomplete (all age groups): 10/13/15/20- PCV, 23-PPSV, hepatitis A, hepatitis B (consider anti-HBs serology), IPV, Hib, dTPa/dTP, MenC/ACWY, MenB, 9vHPV or 4vHPV. In pregnant adolescent: RSV, dTPA booster, inactivated influenza, COVID-19.		

a Dengue vaccine is indicated for adolescents age living in areas with endemic areas.^15^

b Indicated for patients with age 18-years and at risk for Mpox infection.^13^

c **Live-attenuated vaccines:** Generally contraindicated in immunosuppressed patients; however, administration may be considered depending on the degree of immunosuppression, underlying condition, and current treatment. Each case should be individually assessed by the treating physician prior to vaccination. In people living with HIV, immunological status (CD4 T-lymphocyte cell count) should be considered for MMR, varicella and yellow fever vaccination.^12^ *European Centre for Disease Prevention and Control (ECDC) Vaccine Scheduler consolidated national immunization programs across EU/EEA member states. As vaccination policies are defined at the national level, recommendations may vary by country; clinicians are advised to consult their national immunization authority for country-specific guidance.^13^ * In Brazil, the National Program Immunization guides and makes available the vaccines in the public system.^12^^,^^17^ COVID-19: Coronavirus Disease 2019 vaccine dTPa/ dTP, Diphtheria, Tetanus, acellular Pertussis vaccine/Diphtheria, Tetanus, Pertussis vaccine; MenACWY, Meningococcal serogroups A, C, W, Y vaccine; 10/13/15/20- PCV, 10/13/15/20- Pneumococcal Conjugate Vaccine; 23-PPSV, 23- Pneumococcal Polysaccharide Vaccine; MMR, Measles, Mumps, and Rubella vaccine; IPV, Inactivated Poliovirus Vaccine; Hib, Haemophilus influenzae type b vaccine; MenB, Meningococcal serogroup B vaccine; 9vHPV or 4Vhpv, nonavalent or quadrivalent Human Papilomavírus Vaccine; RSV, Respiratory Syncytial Virus prefusion vaccine.

## Declaration of generative AI and AI-assisted technologies in the manuscript preparation process

During the preparation of this work the author(s) used Google Gemini and Claude AI exclusively to improve the English phrasing and clarity of the manuscript. After using this tool/service, the author(s) reviewed and edited the content as needed and take(s) full responsibility for the content of the published article.

## Declaration of competing interest

The authors declare no conflicts of interest.

## Data Availability

The datasets generated and/or analyzed during the current study are available from the corresponding author upon reasonable request.
